# Variations of Growth and Toxin Yield in *Cylindrospermopsis raciborskii* under Different Phosphorus Concentrations

**DOI:** 10.3390/toxins9010013

**Published:** 2016-12-29

**Authors:** Yiming Yang, Yongguang Jiang, Xiaochuang Li, Hua Li, Youxin Chen, Jinlin Xie, Fangfang Cai, Renhui Li

**Affiliations:** 1 Key Laboratory of Algal Biology, Institute of Hydrobiology, Chinese Academy of Sciences, Wuhan 430072, Hubei, China; yangyiming9191@163.com (Y.Y.); xiaochuangli201@gmail.com (X.L.); lih@ihb.ac.cn (H.L.); chenyouxin@ihb.ac.cn (Y.C.); xiejinlin1991@sina.com (J.X.); cai15926359926@163.com (F.C.); 2 Shenzhen Key Laboratory of Marine Bio-Resource and Eco-Environmental Science, Shenzhen University, Shenzhen 518060, Guangdong, China; jyg1949@163.com; 3 University of Chinese Academy of Sciences, Beijing 100049, China; 4 College of Life Sciences, Jiangxi Normal University, Nanchang 330022, Jiangxi, China

**Keywords:** *Cylindrospermopsis raciborskii*, cylindrospermopsin, *cyr* gene, inorganic phosphorus, growth rate

## Abstract

The bloom-forming cyanobacteria, *Cylindrospermopsis raciborskii*, is a producer of the cytotoxic cylindrospermopsin (CYN). In this study, the growth, toxin yield, and expression of CYN biosynthesis genes of *C. raciborskii* were examined under varying phosphorus (P) concentrations. The results show the cell number at 0.00 and 0.01 mg·L^−1^ P was significantly lower than that at higher P concentrations (≥0.5 mg·L^−1^). The chlorophyll *a* content, filament length, heterocyst, and akinete numbers at P ≤ 0.05 mg·L^−1^ were also significantly reduced. The intracellular and extracellular CYN concentrations and the extracellular proportions increased during the culture period, and larger values were observed at higher P concentrations. Total CYN content reached 45.34–63.83 fg·cell^−1^ and extracellular CYN proportion reached 11.49%–20.44% at the stationary growth phase. A significantly positive correlation was observed between CYN production and cell growth rate. Three *cyr* genes were expressed constantly even at P-deficient conditions. The transcription of *cyr* genes at P-replete conditions or after P supplementation increased from 1.18-fold to 8.33-fold. In conclusion, *C. raciborskii* may rapidly reorganize metabolic processes as an adaptive response to environmental P fluctuations. CYN production and *cyr* gene expression were constitutive metabolic processes in toxic *C. raciborskii*.

## 1. Introduction

Increased incidence of harmful cyanobacterial blooms were amongst the most severe environmental problems over the past two decades. Cyanobacterial blooms have attracted considerable scientific attention because of their potential risks related to human sickness and animal mortality and the disruption of aquatic ecosystems [1,2]. Toxic metabolites of cyanobacteria, i.e., cyanotoxins, can be classified into four major categories based on their mode of action. Among these compounds are the hepatotoxic microcystins [3] and nodularins [4]; the cytotoxic cylindrospermopsins (CYNs) [5]; the neurotoxic saxitoxins [6], anatoxin-*a* [7], and beta-methylamino-l-alanine [8]; and the dermatotoxic *Lyngbya* toxins [9] and aplysiatoxins [10]. Specifically, the alkaloid CYN, which is composed of a tricyclic guanidine moiety coupled with a hydroxymethyluracil and a sulfonic acid group, is of particular concern because of the human poisoning event on Palm Island in 1979 [5,11,12]. High concentrations of CYN in water bodies may have been caused by high chemical stability and low degradation efficiency [13]. In addition, CYN can interfere with multiple cellular metabolic processes [14,15] and cause injury in several organs, including the liver, thymus, kidney, and heart [16]. Four natural congeners of CYN have been described, namely, 7-epi-CYN (a C-7 epimer) [17], 7-deoxy-CYN (no hydroxylation on C-7) [18], 7-deoxy-desulfo-CYN and 7-deoxy-desulfo-12-acetyl-CYN [19]. The biosynthesis pathway of CYN has also been proposed based on the deduced function of each CYN gene (*cyr*) [20].

*Cylindrospermopsis raciborskii*, a filamentous diazotrophic cyanobacterial species, belongs to the order Nostocales. As the first recognized CYN producer, *C. raciborskii* has become one of the most well-known bloom-forming cyanobacteria [21,22]. It is emerging in phytoplankton communities worldwide, displaying invasive behaviour as its occurrence spreads from tropical [23] and subtropical [24,25] to temperate climate zones [1,26]. This species is widespread in many regions of the world including Asia, Australia, New Zealand, Europe, Africa, South and North America [27]. However, CYN producers of *C. raciborskii* have so far been reported only in Asian, Australia, and New Zealand water bodies [27]. Several explanations for this phenomenon have been suggested and multiple factors associated with *C. raciborskii* have been proposed [28], including high phenotypic plasticity, wide range of tolerance to key environmental factors [29], superior competition for resources [30], global warming [31], and eutrophication [32]. The relationships among cyanobacterial blooms, nutrient levels, and response of cyanobacteria to changes in nutritional conditions have always been active research topics. Cyanobacteria are frequently subjected to nutrient deficiency, particularly nitrogen, phosphorus (P), and sulfur in natural water bodies [33], which probably affects the physiology and metabolism of these organisms as a consequence. As one of the most essential nutrients, P plays a crucial role in determining primary productivity of water body [34]. Trimbee and Prepas [35] suggested the total P (TP) as a good indicator of the relative biomass of blue-green algae. An alteration in P loading may give rise to a shift in the phytoplankton species composition [36]. Long-term (1991–2013) data in Lake Dianchi showed that variation in the TP concentration had significant effects on the phytoplankton biomass both interannually and seasonally [37]. Chen et al. [38] inferred that the recession of cyanobacterial blooms could arise from the decrease in dissolved phosphorus. As a portion of TP, dissolved P comprised of dissolved inorganic phosphate (DIP) and dissolved organic phosphorus (DOP), can be absorbed and utilized by phytoplankton immediately [39]. DIP is directly bioavailable and is considered the preferred P source for phytoplankton [40] m while DOP can also be exploited [41]. 

Nitrogen is also considered an important nutrient for controlling cyanobacteria growth. However, efforts to reduce nitrogen levels in lakes and reservoirs were accompanied with the increase in the frequency and abundance of Nostocales [42,43]. Most of the reported CYN producers belong to the Nostocales, which can fix atmospheric nitrogen and, thus, compensate for nitrogen limitation [42,43]. Under these circumstances, inorganic P is, therefore, one of the primary restriction factors influencing the growth of CYN-producing species [44]. In a previous study, Bar-Yosef et al. [45] proposed CYN as an allelopathic compound that can induce alkaline phosphatase activity of surrounding phytoplankton to supply inorganic P for *Chrysosporum ovalisporum* (basionym: *Aphanizomenon ovalisporum*). To understand this phenomenon, the distribution of CYN within cyanobacterial cells and freshwater bodies must be clarified first.

Previous studies showed that CYN is highly water-soluble because of its sulfonic acid group and can be largely released into the extracellular environment [46,47]. In German lakes, the cyanobacterial community were mainly composed of *Aphanizomenon*, *Anabaena*, *Anabaenopsis*, *Raphidiopsis*, *Cylindrospermopsis* and *Planktothrix*, and more than 80% of total CYN was detected extracellularly among 31% of the samples [47]. Jiang et al. [48] observed high extracellular percentages of CYN (92%–96%) in *C. raciborskii* AWT 205. In another study with this cyanobacterium, the percentage of extracellular CYN increased from 20% during the rapid growth phase to 50% during the slow growth phase [49]. However, Saker and Griffiths [50] found up to 90% of CYN remained within the cell during exponential growth in seven cultured strains of *C. raciborskii*. In addition, Bormans et al. [51] observed no relationship between CYN concentration and growth rate in benthic species *Oscillatoria* sp. PCC 6506 and the percentage of extracellular CYN varied between 56% and 96%. Moreover, several studies have inferred that the release of CYN increases as cells age, based on observations that the highest dissolved CYN content was found in older blooms [46] or during stationary phase of batch cultures [49,52]. Consistent with these reports, Davis et al. [53] regarded CYN release into the water column as a result of cell lysis during the stationary phase or environmental stress.

Given the critical importance of P availability in growth and blooms development of cyanobacteria, we investigated the growth and toxin yield of a toxic *C. raciborskii* strain under a range of inorganic P concentrations. This study measured the changes in morphology, toxin yield and the expression of *cyr* genes in *C. raciborskii*. The key genes chosen were involved in CYN biosynthesis and transport, such as *cyrA* (amidinotransferase, the first step in CYN biosynthesis), *cyrJ* (sulfotransferase), and *cyrK* (putative transport).

## 2. Results

### 2.1. Effects of P Concentration on the Growth and Morphology of *C. raciborskii*

**Figure 1:**
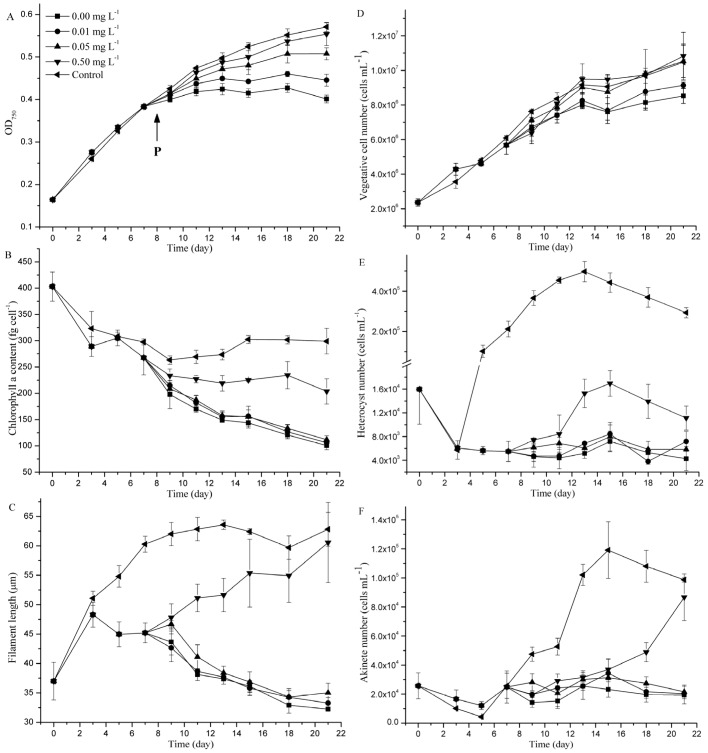
Growth and morphology of *C. raciborskii* incubated with different P concentrations. (**A**) OD_750_ value; (**B**) chlorophyll *a* content; (**C**) filament length; and (**D**–**F**), the number of total cells, heterocysts and akinetes. Error bars represent standard deviations. The vertical arrow shows the time when phosphate was supplemented.

During eight days of P starvation, cell growth rates of P starvation treatment and control group were 0.15 ± 0.03 day^−1^ and 0.17 ± 0.02 day^−1^, respectively, with no significant difference (*p* > 0.05). Moreover, reduced chlorophyll *a* contents and filament lengths and inhibition of heterocyst and akinete formation were observed (Figure 1B,C,E,F). However, in the subsequent culture period, the cell growth rate, chlorophyll *a* content, and morphological parameters were all reduced even after P was supplied at the eighth day, compared with the control group with excess P concentration. Furthermore, the OD_750_ values and the cell number at 0.00 and 0.01 mg·L^−1^ P were significantly lower than those at higher P concentrations (*p* < 0.05; Figure 1A,D). The maximum OD_750_ values were 0.40 ± 0.01, 0.45 ± 0.01, 0.51 ± 0.01, and 0.55 ± 0.03 for 0.00, 0.01, 0.05, and 0.50 mg·L^−1^ P, respectively (Figure 1A). The maximum cell numbers were 8.53 × 10^6^ ± 4.37× 10^5^, 9.13 × 10^6^ ± 1.86× 10^5^, 1.05 × 10^7^ ± 9.36× 10^5^, and 1.08 × 10^7^ ± 1.37 × 10^6^ cells·mL^−1^ for 0.00, 0.01, 0.05, and 0.50 mg·L^−1^ P, respectively (Figure 1D). The maximum OD_750_ value and cell number in the control group were 0.57 ± 0.01 and 1.05 × 10^7^ ± 1.05 × 10^6^ cells·mL^−1^, respectively.

The chlorophyll *a* content (after the 10th day), filament length (after the 10th day), and heterocyst (after the 12th day) and akinete (after the 17th day) numbers at 0.00, 0.01, and 0.05 mg·L^−1^ P were significantly lower than those at 0.50 mg·L^−1^ P and in the control group (*p* < 0.05; Figure 1B,C,E,F). The chlorophyll *a* contents at 0.00, 0.01, and 0.05 mg·L^−1^ P were decreased from 402.89 ± 27.70 fg·cell^−1^ to 100.51 ± 7.45, 106.19 ± 3.45, and 111.86 ± 7.46 fg·cell^−1^, respectively, for each group, compared with the final chlorophyll *a* contents of 203.66 ± 23.99 and 298.95 ± 24.46 fg·cell^−1^ for 0.50 mg·L^−1^ P and the control group, respectively (Figure 1B). Similarly, the filament lengths were decreased from 36.99 ± 3.20 μm to 32.23 ± 0.06, 33.26 ± 0.94, and 35.02 ± 1.63 μm for 0.00, 0.01, and 0.05 mg·L^−1^ P, respectively, whereas the filament lengths for 0.50 mg·L^−1^ P and the control group were greater at 60.56 ± 6.82 and 62.81 ± 2.88 μm, respectively, at the end of the experimental stationary phase (Figure 1C). The maximum numbers of heterocysts were 7.18 × 10^3^ ± 1.73 × 10^3^, 8.42 × 10^3^ ± 1.54 × 10^3^, 7.97 × 10^3^ ± 2.39 × 10^3^, and 1.70 × 10^4^ ± 2.17 × 10^3^ heterocysts mL^−1^ for 0.00, 0.01, 0.05, and 0.50 mg·L^−1^ P, respectively (Figure 1E). The maximum numbers of akinetes were 2.56 × 10^4^ ± 9.23 × 10^3^, 3.43 × 10^4^ ± 9.95 × 10^3^, 3.13 × 10^4^ ± 3.36 × 10^2^, and 8.67 × 10^4^ ± 1.60 × 10^4^ akinetes mL^−1^ for 0.00, 0.01, 0.05, and 0.50 mg·L^−1^ P, respectively (Figure 1F). The maximum heterocyst and akinete numbers in the control group were 4.97 × 10^5^ ± 5.04 × 10^4^ heterocysts mL^−1^ and 1.19 × 10^5^ ± 1.95 × 10^4^ akinetes mL^−1^, respectively.

### 2.2. CYN Measurement

**Figure 2:**
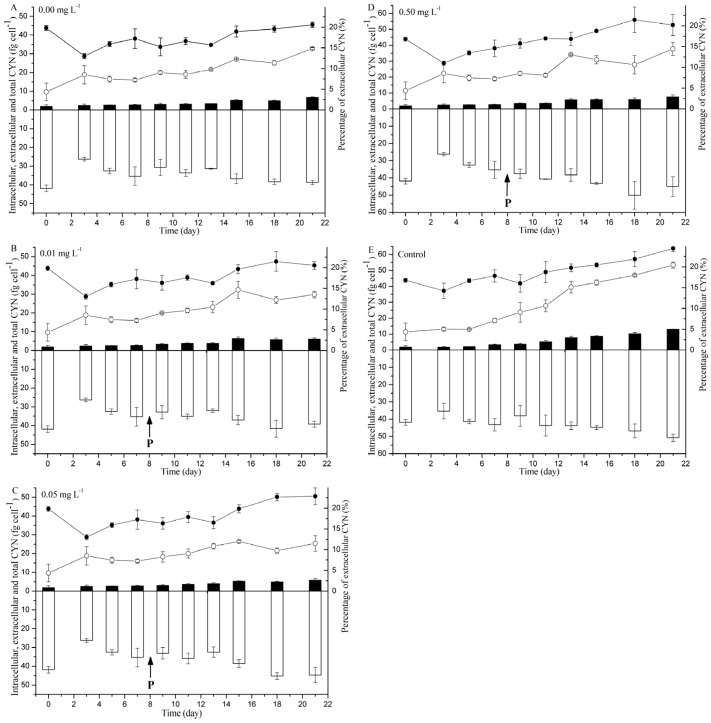
CYN content in *C. raciborskii* culture with P concentrations of (**A**) 0.00 mg·L^−1^, (**B**) 0.01 mg·L^−1^, (**C**) 0.05 mg·L^−1^, (**D**) 0.50 mg·L^−1^ and (**E**) Control. The pool size of intracellular (□), extracellular (■), and total CYN (●), as well as the percentage of extracellular CYN (○) were displayed. Error bars represent standard deviations. The vertical arrows show the time when phosphate was supplemented.

CYN contents in the *C. raciborskii* culture were measured and normalized to the total cell numbers (Figure 2). The cell quotas of intracellular, extracellular, and total CYN gradually increased under all treatments over the experimental period. The total CYN cell quota was calculated as the sum of intracellular and extracellular CYN cell quota. Moreover, the groups with higher P concentrations had larger CYN amounts than the groups with lower P concentrations at most time points. Intracellular CYN accounted for the majority of total CYN with 38.72 ± 1.12, 39.21 ± 1.61, 44.71 ± 4.08, 45.06 ± 5.78, and 50.79 ± 2.13 fg·cell^−1^ for P concentrations of 0.00, 0.01, 0.05, and 0.50 mg·L^−1^ and the control group, respectively (Figure 2). A minor portion of CYN was extracellular with 6.75 ± 0.32, 6.13 ± 0.66, 5.80 ± 1.00 and 7.61 ± 1.16 fg·cell^−1^ for cells grown in P concentrations of 0.00, 0.01, 0.05, and 0.50 mg·L^−1^, which were significantly lower than 13.04 ± 0.14 fg·cell^−1^ in the control group (*p* < 0.01; Figure 2). The final total CYN contents reached 45.47 ± 1.43, 45.34 ± 2.23, 50.51 ± 4.43, 52.67 ± 6.59, and 63.83 ± 2.09 fg·cell^−1^ for P concentrations of 0.00, 0.01, 0.05, and 0.50 mg·L^−1^ and the control group, respectively (Figure 2). With regard to intracellular CYN and total CYN, no significant differences were found among cells grown with P ≤ 0.05 mg·L^−1^ (*p* > 0.05), while the values of cells grown with P > 0.05 mg·L^−1^ were significantly higher than that of those under P depletion condition (*p* < 0.01).

**Figure 3:**
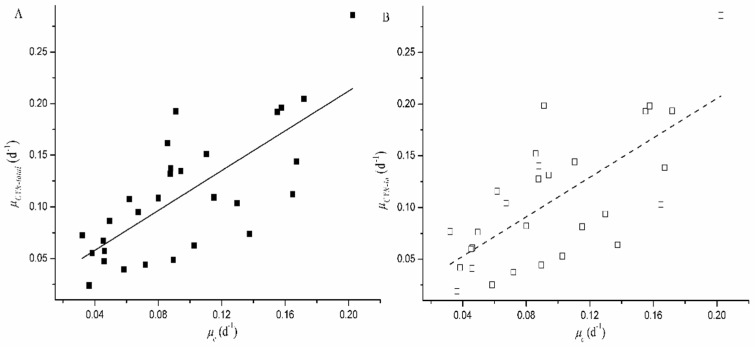
Correlations between specific toxin production rate (*μ_CYN_*) and the corresponding specific growth rate (*μ_c_*) during culture period. (**A**) *μ_CYN-total_* represents specific total toxin production rate and (**B**) *μ_CYN-in_* represents specific intracellular toxin production rate. The linear regression equations are as follows: (**A**) *y* = 0.96*x* + 0.02, *R*^2^ = 0.54, *p* = 0.00; and (**B**) *y* = 0.95*x* + 0.01, *R*^2^ = 0.48, *p* = 0.00.

The percentage of extracellular CYN also increased during the sampling period. At the last day of the experiment, a significantly higher CYN percentage was observed to be extracellular in the control group (20.44% ± 0.75%), compared with extracellular percentages of 14.83% ± 0.23%, 13.50% ± 0.81%, 11.49% ± 1.86%, and 14.45% ± 1.60% at P concentrations of 0.00, 0.01, 0.05, and 0.50 mg·L^−1^, respectively (*p* < 0.01; Figure 2). Furthermore, both *μ_CYN__-total_* and *μ_CYN__-in_* showed a significantly positive correlation with *μ_c_* (*p* < 0.05; Figure 3). The slope were 0.96 and 0.95, respectively, both of which were not significantly different from 1.00 (ANCOVA: *p* = 0.83 for *μ_CYN__-total_* and *p* = 0.80 for *μ_CYN__-in_*).

### 2.3. cyr Gene Expression

**Figure 4:**
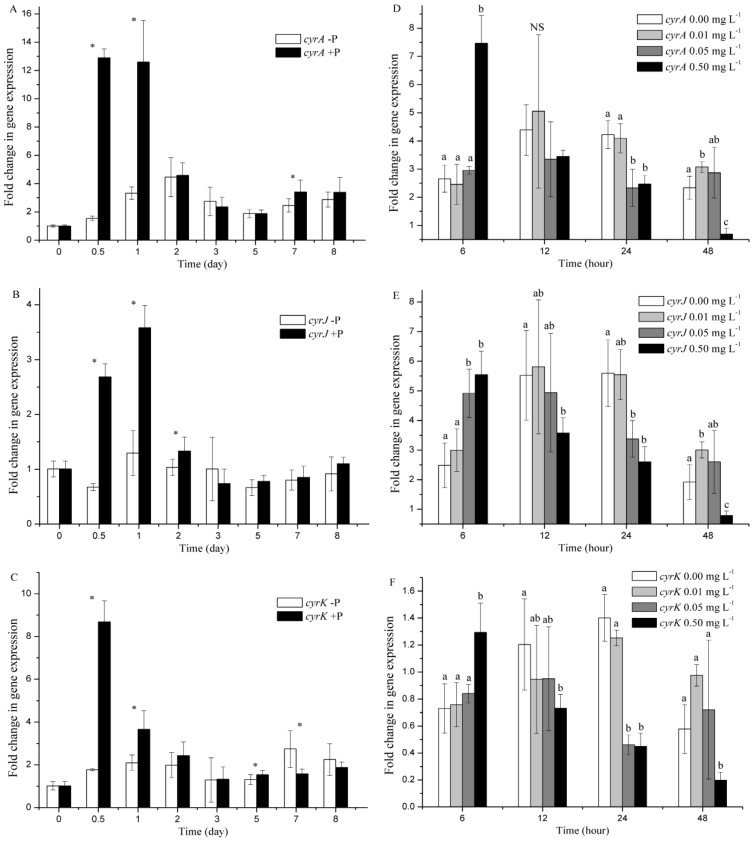
Relative expression levels of *cyr* genes under P starvation (**A**–**C**) and varying P concentrations (**D**–**F**). Fold changes in gene expression were calculated by dividing the values at time = 0. The time in **D**, **E**, and **F** was counted after the supplement of P. Error bars represent standard deviations. Asterisk and different letters represent significant differences among groups (*p* < 0.05) at a single time point.

The expression levels of three *cyr* genes, namely, *cyrA*, *cyrJ*, and *cyrK*, were examined, and their transcript abundances were expressed as the fold changes with respect to time = 0. In the P starvation stage, upregulated expression levels of *cyrA* and *cyrK* genes were observed for the control and experimental groups compared with the initial expression levels (Figure 4A,C). The expression of *cyrJ* gene was also increased in the early stage of P starvation and was reduced to initial levels subsequently (Figure 4B). Furthermore, the expression levels of *cyrA* gene in the P-depleted group were 12%, 26%, and 72% of those in the control group after 0.5, 1, and 7 days, respectively, with significant difference (*p* < 0.05). The expression levels of *cyrJ* gene in the P-depleted group were 25%, 36%, and 77% of those in the control group after 0.5, 1, and 2 days, respectively, with significant difference (*p* < 0.05). The expression levels of *cyrK* gene in the P-depleted group were 20%, 57%, and 85% of those in the control group after 0.5, 1, and 5 days, respectively, with significant difference (*p* < 0.05). However, a significant 1.74-fold increase in *cyrK* gene expression was observed in the P-depleted group after seven days compared with the control group. Moreover, no significant difference was observed for the expression levels of *cyr* genes between P-depleted and control groups at the last day of P starvation (*p* > 0.05).

After P supplementation on the eighth day, the expression levels of *cyrA* and *cyrK* genes were significantly increased to 2.81-fold and 1.77-fold, respectively, at 0.50 mg·L^−1^ P compared with those in the P-depleted group after 6 h (*p* < 0.05; Figure 4D,F). Similarly, the expression levels of *cyrJ* gene at 0.05 and 0.50 mg·L^−1^ P were 1.97-fold and 2.23-fold higher, respectively, than those in the P-depleted group after 6 h, with significant difference (*p* < 0.05; Figure 4E). Afterward, the *cyr* gene expression levels at 0.05 mg·L^−1^ P were reduced and were 55%, 60%, and 33% of those in the P-depleted groups for the *cyrA*, *cyrJ*, and *cyrK* genes, respectively, after 24 h, with significant difference (*p* < 0.05). The *cyr* gene expression levels at 0.50 mg·L^−1^ P were also reduced and were 30%–79%, 41%–65%, and 32%–61% of those in the P-depleted group for the *cyrA*, *cyrJ*, and *cyrK* genes, respectively, with significant difference, except after 12 h. The *cyr* gene expression levels at 0.01 mg·L^−1^ P were not significantly different from that in the P-depleted group at most time points, except for significantly higher expression levels of the *cyrA* and *cyrJ* genes at 0.01 mg·L^−1^ P after 48 h.

### 2.4. Correlation between the Biomass of Cylindrospermopsis and TP Concentration in Freshwater Bodies

**Figure 5:**
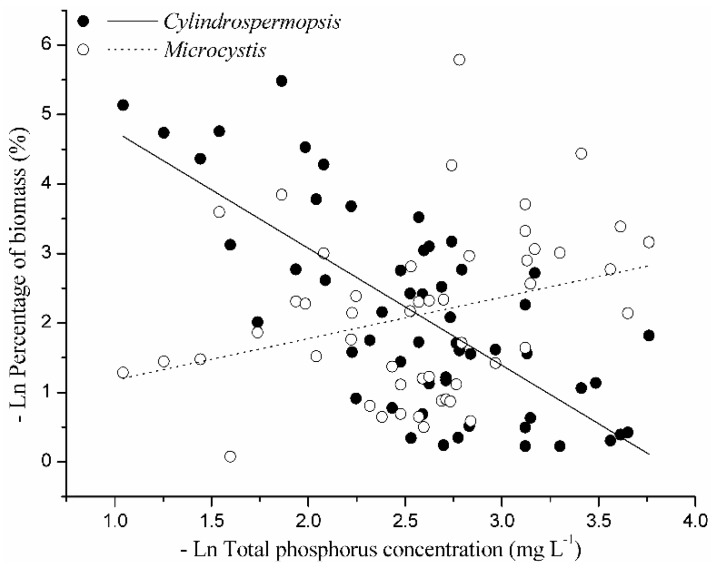
Correlations between percentage of cyanobacterial biomass and TP concentrations in freshwater bodies. *Cylindrospermopsis*, *y* = −1.68*x* + 6.44, *R*^2^ = 0.50, *p* = 0.00; *Microcystis*, *y* = 0.60*x* + 0.58, *R*^2^ = 0.08, *p* = 0.03.

Among a series of phytoplankton species and water quality parameters, we centered on *Cylindrospermopsis* and TP concentration. The percentage of *Cylindrospermopsis* in total cyanobacterial biomass showed the tendency of negative correlation with TP concentration (*y* = −1.68*x* + 6.44, *R*^2^ = 0.50, *p* = 0.00), compared with the positive correlation between the percentage of *Microcystis* and TP concentration (*y* = 0.60*x* + 0.58, *R*^2^ = 0.08, *p* = 0.03) (Figure 5).

## 3. Discussion

In this study, we explored the P-dependent growth, toxin yield, and the expression levels of the *cyr* genes *cyrA*, *cyrJ*, and *cyrK*, in *C. raciborskii* CHAB3438. This strain could grow at very low P concentrations during the experimental period, and this corresponds with the high P affinity and large P storage that has been previously reported for *C. raciborskii* [54,55,56]. However, the chlorophyll *a* content per cell was lower at the lower P concentrations compared to the higher P concentrations. Additionally, filament length and formation of heterocysts and akinetes were also reduced at low P concentrations. The explanation for this physiological response is that *C. raciborskii* is able to economize resources by adjusting metabolic pathways and reducing high energy-consuming physiological processes under limited nutrient supply, a response also found in *Microcystis aeruginosa* [57]. The phenotypic plasticity of multiple traits may allow *C. raciborskii* to optimize growth in P fluctuations, which may explain its dominance in the phytoplankton of many aquatic ecosystems, particularly those with low P concentrations [58]. Furthermore, field surveys of this study also showed a trend where the relative abundance of *Cylindrospermopsis* increased with the decline of TP concentration in lakes. This result is consistent with the investigation of Bonilla et al. [59] that observed a high biomass of *C. raciborskii* under low TP concentration. Therefore, *Cylindrospermopsis* is highly competitive and adaptive under low P concentrations compared with other bloom-forming cyanobacteria, which significantly promotes its invasive spread.

Previous investigations revealed that P deprivation inhibited CYN production in *Chr. ovalisporum* [60]. On the other hand, replete P supply was found to boost CYN yield in *C. raciborskii* and cyanobacterial blooms samples [61,62]. However, only intracellular CYN was measured in these studies, and the extracellular portion of CYN was ignored. On the contrary, an increase in the total CYN content was reported under P-depleted conditions, coupled with upregulated expression levels of the CYN biosynthesis genes [45,63]. Therefore, it is necessary to explore the extracellular CYN concentration under different P concentrations to clarify the contradiction of these results. In this study, the concentrations of intracellular, extracellular and total CYN, as well as the extracellular proportion of CYN, all increased during the culture time and at all P concentrations. The CYN production rate exhibited a positive correlation with cell growth rate, therefore, CYN production is constitutive in *C. raciborskii* CHAB3438. The effects of P concentrations on these processes were produced through affecting cell growth. Therefore, CYN yield would be reduced when cell maximum biomass is reduced under P-limited conditions. These results were supported by previous findings that the CYN pool size of *C. raciborskii* was constant under different nitrogen, phosphorus, light, and CO_2_ conditions [64,65].

At the end of the experiment, the proportion of extracellular CYN reached 11.49% to 20.44% of the total CYN, indicating that CYN is mainly stored in cyanobacterial cells, whereas only a minor proportion of CYN is released into the extracellular environment, consistent with the results of Orr et al. [66] with field cyanobacterial samples. Cell lysis may contribute to extracellular CYN accumulation during the stationary phase as suggested in previous research [53]. In addition, active CYN release from cyanobacterial cells has been emphasized in other studies [63], but it remains to be proved. The hypothetical CYN transporter CyrK has been deduced from the biosynthesis cluster [20] but its function needs to be confirmed by examining a mutant strain lacking the *cyrK* gene.

The transcription of three *cyr* genes that are involved in CYN biosynthesis and transport was examined to better understand the potential response of *cyr* gene cluster in *C. raciborskii* to various P concentrations. The results showed significantly high expression levels of *cyr* genes under P-replete conditions in the early stage of P starvation and in the initial 6 h of P supplementation thereafter. Afterwards, the expression levels of *cyr* genes at various P concentrations maintained similar levels during the experimental period; however, significantly lower expression levels were observed for 0.05 and 0.50 mg·L^−1^ P in comparison to other P concentrations at several time points. Although the *cyr* gene expression did not completely coincide with the production and release of CYN, which was constitutive as aforementioned, the constant expression of *cyr* genes further confirmed CYN as a constitutive metabolite of the toxic *C. raciborskii*. In a study with *C. raciborskii* CS-505, Stucken et al. [67] investigated the relationship between expression levels of four *cyr* genes and CYN production in response to nitrogen source. They also found that these genes showed a constitutive transcription and concluded that CYN biosynthesis was not regulated at the transcriptomic level. Furthermore, Pierangelini et al. [64] reported differential expression of *cyr* genes in contrast to constant CYN cell quotas in different growth stages of *C. raciborskii*, and suggested CYN biosynthesis was post-transcriptionally regulated. Our results support the post-transcriptional regulation of CYN for a constant cell quota.

## 4. Conclusions

In summary, the morphology of *C. raciborskii* and the expression of the genes *cyrA, cyrJ*, and *cyrK*, altered in response to variations in environmental P, however, CYN production and release remain constitutive processes. The majority of CYN occurred intracellularly with only a small proportion (4.38%–14.83%) occurring extracellularly. Finally, the toxin yield of *C. raciborskii* blooms would be reduced with a reduction in available P, as P limits the maximum total cell number.

## 5. Materials and Methods

### 5.1. Strain and Culture Conditions

CYN-producing strain *C. raciborskii* CHAB3438 was previously isolated from Lake Xianghu and maintained in liquid MA medium [68]. Quintuplicate 1 L pure cultures in sterile 2 L Erlenmeyer flasks were incubated in 25 °C with a light intensity of 35 μmol m^−2^·s^−1^ (12/12 h light/dark) provided by cool white fluorescent tubes. The cultures were manually shaken thrice daily during incubation until the exponential phase.

### 5.2. Phosphorus Starvation and Supplementation

The cultures were filtered using Millipore membranes (3.0 μm pore size, Merck Millipore, Darmstadt, Germany), washed thrice with sterile P-free MA medium, resuspended, and subcultured into five groups. Each group included triplicate cultures containing 50 mL concentrated cultures and 1150 mL P-free MA medium (four experimental groups) or full medium (one control group with 10 mg·L^−1^ P). The starting cell concentration was 2.36 × 10^6^ ± 2.10 × 10^5^ cells·mL^−1^. The cultures were incubated for eight days to deplete intracellular polyphosphate stores. Afterward, the four experimental groups were supplemented with K_2_HPO_4_ solution to obtain the final P concentrations of 0.00, 0.01, 0.05, and 0.50 mg·L^−1^. Fresh media with corresponding P concentrations were supplemented after each sampling to maintain constant culture volumes. Samples were collected on alternate days to determine cyanobacterial growth, CYN content, and gene expression level. In the P starvation stage, three of the 12 experimental cultures were randomly selected to measure each parameter. Therefore, the data of growth and CYN are shared by four experimental groups during the first eight days of P starvation.

### 5.3. Measurement of Growth

Growth was measured spectrophotometrically at 750 nm. Subsamples (1 mL) were preserved in Lugol’s iodine solution for microscopic measurement (Olympus BX51, 400× magnification, Olympus, Tokyo, Japan) of morphological features, including cell concentration, heterocyst and akinete numbers, and filament length. Cell concentration was measured as described in Pierangelini et al. [69]. Filament length measurements were performed on at least 100 trichomes. For chlorophyll *a* measurement, 20 mL of cell culture was filtered through a glass fiber filter (47 mm diameter, 1.2 μm pore size; Whatman GF/C) and extracted using 90% acetone solution in the dark for 24 h at 4 °C. The extracts were centrifuged at 8000 rpm for 30 min, and the absorbance of the supernatant was measured at 750, 663, 645, and 630 nm. The concentration of chlorophyll *a* was calculated using the formula applied by the China Environmental Protection Administration [70].

### 5.4. Extraction and Quantitation of CYN

A cell culture volume of 25–50 mL was filtered by Millipore filters (0.22 μm pore size). Intracellular and extracellular CYN were extracted from filters and filtrates, respectively. The collected cells on the filters were resuspended in 1 mL distilled water, fractured by a SPEX 6870 Freezer Mill for 10 min, shaken for 1 h at room temperature, and centrifuged at 12,000 rpm for 30 min. The supernatants were collected and combined after the extraction step was repeated. The supernatants and filtrates were subjected to solid-phase extraction to enrich CYN, as described previously [71]. Afterward, the elutes were further concentrated by rotary evaporation. The precipitate was redissolved in 0.5 mL of Millipore water and filtered through an ultra-centrifugal filter (10 kDa). CYN was detected by high-performance liquid chromatography, and the column and elution conditions were set as descried by Jiang et al. [48]. Standard CYN (Enzo Life Sciences, New York, NY, USA) was used for qualitative and quantitative analyses. CYN concentrations in the samples were calculated based on UV absorbance at 262 nm. The specific growth rate (*μ_c_*) and specific toxin production rate (*μ_CYN_*) were calculated using the equation:

(1) $$\mu \;=\frac{{\ln C}_{2}-{lnC}_{1}}{t_{2}\;-t_{1}},$$

where *C*_1_ and *C*_2_ are the cell number (cells·mL^−1^) and CYN concentration (μg·CYN·L^−1^) at times *t*_1_ and *t*_2_, respectively [64]. Data of days 3–21 were used to calculate *μ_c_* and *μ_CYN_*. The slope of regression curves were statistically analyzed, as described previously [67].

### 5.5. RNA Isolation and cDNA Synthesis

Cells were collected via centrifugation of 9.0–40 mL of culture at 8000 rpm, 4 °C for 2 min, and total RNA was extracted using the RNAiso Plus Kit (Takara, Otsu, Japan) according to the manufacturer’s instructions. DNA digestion was performed after RNA isolation with RNase-free DNase (Promega, Madison, WI, USA) and RNase Inhibitor (Thermo, Waltham, MA, USA). RNA quality and purity was assessed using a Nanodrop spectrophotometer (Thermo, Waltham, MA, USA) and a total of 900 ng of RNA was taken for reverse transcription (RT) reaction using the PrimeScript™ RT reagent Kit with gDNA Eraser (Takara, Otsu, Japan) according to the manufacturer’s instructions. Genomic DNA was further removed with gDNA Eraser during this process. The absence of genomic DNA in the RNA samples was then verified by a RT-PCR control containing no reverse transcriptase.

### 5.6. Quantitative PCR (qPCR) Assay

A three-step qPCR programme was performed in an iCycler iQ5 (Bio-Rad, Hercules, CA, USA). The procedure consisted of 95 °C for 1 min, followed by 40 cycles of 95 °C for 15 s, 55 °C for 15 s, 72 °C for 45 s and then a melt-curve cycle of 0.5 °C increases at 15 s intervals to verify the quality of the product. All reactions were run in triplicate, which comprised 10 μL 2× THUNDERBIRD SYBR qPCR Mix (Toyobo, Osaka, Japan), 0.2 μM forward and reverse primers, 2 μL cDNA and sterile Millipore water to a final reaction volume of 20 μL. Sanger sequencing was performed on the amplicons to confirm the presence of *cyr* gene fragments. The qPCR primers were designed according to *cyr* gene sequences of *C. raciborskii* CHAB3438 (GenBank accession number: KJ139743). Standard curve of each primer pair was established by 10-fold dilutions of a PCR template. The qPCR primer sequences, amplicon sizes and amplification efficiencies are shown in Table 1. The time = 0 point sample was used as a calibrator and 16S rDNA was chosen as a reference gene for calculating fold changes of *cyrA*, *cyrJ* and *cyrK* genes using the 2^−ΔΔ*Ct*^ method, where

ΔΔ*C_t_* = (*C_t_*_, target gene_ − *C_t_*_, 16S *rrn*_)*_t_* − (*C_t_*_, target gene_ − *C_t_*_, 16S *rrn*_)*_t_*_= 0_. (2)

### 5.7. Collection of Water Samples

A total of 55 sampling sites belonging to 23 shallow lakes were covered in this study. These water bodies were located in the east of China and water samples were collected in the summer of 2013 (Table S1). Analysis of water quality parameters and phytoplankton species composition were conducted as previously described [72].

### 5.8. Statistical Analysis

Data are presented as the mean ± standard deviation. Statistical difference was tested by one-way ANOVA and Tukey’s post hoc comparison test implemented in SPSS v19.0 software (IBM, Chicago, IL, USA) for Windows. Differences with *p* values less than 0.05 were considered significant.

## Figures and Tables

**Table 1:** Characteristics of primer pairs used in RT-qPCR reactions.

Gene	Primer	Sequence (5′–3′)	Product Size (bp)	Efficiency (%)
*cyrA*	qcyrAF193	GAGGAGTTGAATGGGCTGGTA	136	98.94
	qcyrAR328	GTGGGCAGACCGCACAATA		
*cyrJ*	qcyrJF375	TCTGATTCGCCAACCCAAAG	133	98.63
	qcyrJR507	CGGGATTACTCCGCTCGTT		
*cyrK*	qcyrKF345	CGGGAAATAGCCAACACG	106	102.02
	qcyrKR450	AAAGGGAAAGGAGCCACA		
16S rDNA	q16SF1029	GTGTCGTGAGATGTTGGGTT	182	101.17
	q16SR1210	CCTCTGTCCGTAGCATTGTAG		

## Supplementary Materials

The following are available online at www.mdpi.com/2072-6651/9/1/13/s1, Table S1: Details of field samples.
